# Machine Learning-Driven Prediction of Vitamin D Deficiency Severity with Hybrid Optimization

**DOI:** 10.3390/bioengineering12020200

**Published:** 2025-02-18

**Authors:** Usharani Bhimavarapu, Gopi Battineni, Nalini Chintalapudi

**Affiliations:** 1 Department of Computer Science and Engineering, Koneru Lakshmaiah Education Foundation, Vaddeswaram 522302, India; ushafdp1122@gmail.com; 2 Clinical Research Centre, School of Medicinal and Health Products Sciences, University of Camerino, 62032 Camerino, Italy; gopi.battineni@unicam.it; 3 The Research Centre of the ECE Department, VR Siddartha Deemed University, Vijayawada 521002, India

**Keywords:** vitamin D, stacking classifier, whale optimization, performance metrics

## Abstract

There is a growing need to predict the severity of vitamin D deficiency (VDD) through non-invasive methods due to its significant global health concerns. For vitamin D-level assessments, the 25-hydroxy vitamin D (25-OH-D) blood test is the standard, but it is often not a practical test. This study is focused on developing a machine learning (ML) model that is clinically acceptable for accurately detecting vitamin D status and eliminates the need for 25-OH-D determination while addressing overfitting. To enhance the capacity of the classification system to predict multiple classes, preprocessing procedures such as data reduction, cleaning, and transformation were used on the raw vitamin D dataset. The improved whale optimization (IWOA) algorithm was used for feature selection, which optimized weight functions to improve prediction accuracy. To gauge the effectiveness of the proposed IWOA algorithm, evaluation metrics like precision, accuracy, recall, and F1-score were used. The results showed a 99.4% accuracy, demonstrating that the proposed method outperformed the others. A comparative analysis demonstrated that the stacking classifier was the superior choice over the other classifiers, highlighting its effectiveness and robustness in detecting deficiencies. Incorporating advanced optimization techniques, the proposed method’s promise for generating accurate predictions is highlighted in the study.

## 1. Introduction

The growth, development, and overall well-being of the body is dependent on vitamins and minerals, which are crucial for its growth, development, and well-being [1]. The body is unable to synthesize vitamins by itself, so they are usually acquired through dietary intake and supplementation. The two main categories of vitamins are fat-soluble (i.e., A, D, E, and K) and water-soluble (i.e., C and B-complex), and it is worth highlighting that specific vitamins can be produced in restricted amounts by converting provitamins [2]. Exposure to ultraviolet rays or sunlight is a way to synthesize vitamin D from provitamin D [3]. There are several important roles that vitamin D plays in maintaining human health, including its impact on gene expression, modulating the immune system, regulating inflammation, influencing apoptosis, and promoting blood vessel growth [4]. Vitamin D2 and vitamin D3 are fat-soluble molecules with two distinct forms [5].

Various diseases, such as cardiovascular diseases [6], hypertension [7], diabetes mellitus [8], and certain cancers [9], have been linked to low levels of vitamin D. Adults over the age of 70 should aim for a daily intake range of 800 to 1000 IU [10]. These guidelines promote overall health and well-being, as vitamin D is crucial in various bodily functions, including bone health and immune system support [11]. The public health problem of vitamin D deficiency (VDD) is widespread and has significant variations across different populations and regions. Approximately 15.7% of individuals worldwide have serum 25-hydroxyvitamin D levels below 30 nmol/L, a threshold commonly used to define deficiency [12]. Certain groups have a higher prevalence in the United States, with 41.6% of adults deficient, with Black (82.1%) and Hispanic (69.2%) populations being the most affected [13]. Limiting sun exposure, higher latitudes, eating habits, skin pigmentation, and socioeconomic status all play roles in causing these disparities.

In recent years, there has been a significant increase in the number of vitamin D tests conducted globally, which reflects the growing awareness of its importance [14]. The recommendation to screen the entire population for VDD is not recommended due to the high cost of measuring serum 25(OH)D [15]. To reduce indirect health costs, innovative strategies like guided blood tests or target testing are necessary, with a focus on individuals at high risk of VDD or insufficiency [16]. The challenge is to accurately identify these individuals. Therefore, creating a screening tool to evaluate people before suggesting vitamin D testing could be useful in minimizing unnecessary tests and reducing their associated costs.

VDD severity has been assessed using questionnaires and statistical models, namely Linear Regression (LR), Multivariable Adaptive Regression Spline (MARS), and Support Vector Machines (SVM) with regression [17,18]. In the past, outcomes were typically compared among various statistical models, with the most common use of LR being severity prediction. Due to its limitations in predictive performance and managing multiple parameters, LR’s effectiveness was limited. The use of machine learning algorithms for severity prediction has not been explored in past research.

The current challenge of high expenses associated with VDD assessments is caused by the traditional reliance on biochemical methods for VDD analysis. Given the research gap, it is imperative to streamline the complex analytical procedures for identifying patient VDD. To address this matter, our study proposes a model that can predict the severity of VDD among patients. The Improved Whale Optimization Algorithm (IWOA) is used to select the most pertinent features for detecting VDD by optimizing the feature subset for classification tasks [19]. Compared to conventional WOA, the hybrid approach of IWOA is more effective because traditional WOA can sometimes suffer from premature convergence or inefficient exploration [20]. By employing a parallel search process, IWOA addresses these issues and achieves better global exploration while refining results through local search. As a result, IWOA achieves faster convergence and better feature selection by effectively balancing exploration of new solutions with exploitation of the best solutions so far. With local search, IWOA avoids the limitations of WOA models by selecting sparse yet highly discriminative features, improving VDD detection accuracy.

An Improved Whale Optimization Algorithm (IWOA) is proposed to select optimal features, aiming to enhance accuracy. This paper contributes novel insights into identifying and categorizing VDD severity, paving the way for more effective and efficient diagnostic approaches.

## 2. Materials and Methods

This section introduced various methodological approaches, which encompass data collection, data preprocessing, feature selection using the IWOA algorithm, and data classification by a stacking classifier.

### 2.1. Data Collection

Demographic data were collected from the participants, including their age, gender, reported medical history, and medication usage. The availability of sunlight is a major factor in influencing vitamin D synthesis, and it fluctuates across different geographic locations and seasons. Our investigation focused on geographic factors, particularly those from Vijayawada, Andhra Pradesh, to account for regional variations in sunlight exposure. Moreover, longitudinal data that monitored vitamin D levels of the same individuals over multiple seasons were gathered. We employed the 11-item Food Diversity Score Kyoto (FDSK-11) for food-intake evaluation [21]. According to the VIDSUN questionnaire, participants provided information about sun exposure, vitamin D supplement use, and regular fish consumption [22]. Fish containing high levels of vitamin D were defined as those with over 10 μg of vitamin D in the habitual intake value of the diet [23]. The four primary groups defining vitamin D levels are Optimal, Suboptimal, Inadequate, and Extremely Inadequate. Table 1 lists all 12 parameters we utilized in this study.

A dataset with responses from 512 participants was compiled between 2022 and 2023 in a comprehensive manner. By filling out questionnaires and submitting test reports, these individuals gained insight into their health and personal choices. Incorporating diverse sources like hospital records, medical imaging (X-rays and scans), and doctors’ prescriptions, our data collection process utilized a holistic approach. Aside from clinical data, the dataset also incorporates user preferences, such as lifestyle factors such as diet, exercise, and other health-related choices. The extraction of medical information and personal inclinations was performed through the analysis of textual data. The participant frequency at the VDD levels is shown in Table 2. VDD levels are established on medical standards that are recognized. The Optimal levels are above 30 ng/mL, the Suboptimal levels are between 20 and 30 ng/mL, the Inadequate levels are between 10 and 20 ng/mL, and the Extremely Inadequate levels are below 10 ng/mL. Organizations like the Institute of Medicine (IOM) and the Endocrine Society establish these thresholds, which are then used to predict and classify vitamin D sufficiency in individuals. Specific clinical guidelines or regional practices may influence the exact numerical thresholds.

### 2.2. Data Preprocessing

Data preprocessing is a crucial part of the ML pipeline, particularly when dealing with real-world datasets. Missing data are addressed using Multiple Imputation by Chained Equations (MICE), a method well-suited for datasets where the missingness is random. MICE create multiple imputed datasets by using observed data to predict missing values [24]. When categorical variables were missing, the mode was used, and continuous variables, such as age and body mass index, were imputed based on their relationships. Outliers were identified using the Interquartile Range (IQR) method, where values falling outside the range of 1.5 times the IQR above the 75th percentile or below the 25th percentile were considered outliers. These outliers were either capped to the boundary values or removed if they were deemed extreme and non-representative of the population.

Continuous features were normalized to ensure that all features have a comparable scale through Z-score standardization [25]. To reduce noise and variability, some data points were smoothed using aggregation methods. For instance, fish intake and milk consumption were aggregated based on participant-reported dietary patterns, simplifying the categories and making them easier to interpret. Additionally, the Calcium Intake Score was transformed into categories (e.g., >16, 1–15, and 0–7) to represent different levels of intake more clearly. These transformations allowed for easier interpretation and better model performance [26].

The dataset features were transformed to match the proposed ML model using hot and ordinal encoding techniques. We used the Synthetic Minority Over-sampling Technique (SMOTE-NC) to address class imbalance in this dataset, which includes both categorical and continuous features. The dataset contains a continuous variable named ‘FDSK Score’, while the rest are categorical. Synthetic samples for minority classes are generated by SMOTE-NC using interpolation between existing instances and choosing the most frequent category from their nearest neighbors.

### 2.3. Feature Selection with IWOA

In this paper, we employed IWOA for feature selection because it works by initially encoding features in a binary format, where each feature is either included or excluded from the subset. The algorithm uses a population of candidate solutions (whales), which represent different combinations of selected features. Through the global search mechanism, inspired by the humpback whale’s bubble-net hunting technique, IWOA explores the solution space to identify potential feature subsets. This phase helps avoid local optima by using both exploration and exploitation strategies. However, while WOA can converge quickly to a solution, it may lack the fine-grained refinement needed for optimal feature selection. To address this, IWOA introduces a memetic refinement process, typically using Simulated Annealing, to further refine the candidate solutions. This local search mechanism makes incremental adjustments to feature subsets, optimizing the trade-off between classification accuracy and feature subset size, and gradually improves the selected features’ performance.

The WOA’s performance was optimized by adopting a mutation-based Memetic Hyper-Heuristic, which can benefit from the capabilities of global and local search processes by enhancing its performance. The WOA mechanism may converge faster to a satisfactory solution if it draws inspiration from the bio hunt behavior of humpback whales, that of spreading bubbles through the water in a bubble-net, but WOA lacks fine-grained refinements and may be prone to premature termination. With these means and a memetic mechanism, the candidate solution can be further refined, convergent to an optimal solution that quickly approaches or is close to it, using a local search mechanism (such as Simulated Annealing or tabu search).

Clinically, applying Memetic Hyper-Heuristic versus conventional WOA has meaningful benefits for feature selection [27]. The optimization process is much faster and more accurate at the beginning due to the faster and more accurate convergence of Memetic Hyper-Heuristic. As compared to traditional WOA, using local refinement schemes increases the solution accuracy and decreases the convergence time. In addition, a parallel search sequence frees the algorithm from the local optima trap, which is a common optimization problem. By using “global search” and “local search” together in the form of WOA and memetic refinement, an effective global search process is guaranteed. It has emerged as a strong solution when applied to non-trivial problems, including those related to feature selection, because it is highly versatile and highly tunable. The trade-off between exploration and exploitation strategies is responsible for the accuracy of the optimized feature subset. The result is that the extracted feature subsamples not only become sparser but also remain.

During the feature selection phase, the most discriminative features of the dataset are selected to distinguish vitamin D levels. WOA’s binary encoding of the training input is carried over to the feature set encodings, X = [$x_{1},x_{2}\ldots .x_{n}$] is a binary vector, and each feature is included ($x_{i}=1$) or excluded ($x_{i}=0$) of the ith feature. Algorithm 1 defines the step-by-step of IWOA used in this study.


**Algorithm 1**: Memetic Hyper-Heuristic with Whale Optimization for Feature Selection**Input:** Preprocessed dataset with n features. **Output:** Optimized feature subset.**Step 1: Initialization**▪$\mathrm{Randomly}\;\mathrm{initialize}\;a\;\mathrm{population}\;\mathrm{of}\;N\;\mathrm{whales}\;(\mathrm{feature}\;\mathrm{subsets})\;\mathrm{in}\;\mathrm{that}\;\mathrm{each}\;\mathrm{whale},\;\mathrm{where}\;X=[x_{1},x_{2}\ldots .x_{n}$], where $x_{i}$ ∈ {0,1} indicates whether the i-th feature is included (11) or excluded (00).▪Evaluate the fitness of each whale (feature subset) *X* by training the classifier on the selected features and computing its performance using an appropriate metric (e.g., classification accuracy and F1-score).▪Set the best position of the population to the position of the whale with the highest fitness value $X^{*}$(0), i.e., whale with the highest fitness value at iteration 0**Step 2: Whale Optimization Algorithm (WOA)** For each iteration *t* = 1, 2, …, T_max_:  ○For each whale *X*(*t*), update its position using either the exploitation phase (shrinking encircling mechanism) or the exploration phase (spiral updating mechanism)Exploitation phase (bubble-net hunting): *X*(*t* + 1) = $X^{*\;}\left(t\right)-A\cdot \;\left|C\cdot X^{*\;}(t)-X(t)\right|$Exploration phase (spiral movement): *X*(*t* + 1) = $\left|{D\cdot X}^{*\;}(t)-X(t)\right|\cdot e^{bl}\cdot \cos\left(2\pi l\right)+X^{*\;}(t)$  ○Convert the continuous whale positions to binary values using a sigmoid function:
$x_{i}(t+1)$ =$\;\left\{\begin{array}{c} 1\;if\;S(x_{i})>rand\\ 0\;\;\;\;\;\;\;\;\;\;\;otherwise \end{array}\right.$
where S ($x_{i}$) = $\frac{1}{1+e^{x_{i}(t+1)}}$.  ○Evaluate the fitness of the updated whale positions using the same classifier and metric as in Step 1.  ○If a whale has better fitness than the current best, update the best position:  ○$X^{*}$(*t* + 1) = whale with highest fitness value at iteration *t* + 1.**Step 3: Memetic Refinement (Local Search)** For each whale at the end of the global search, perfom the following:
  ○Apply a local refinement technique like Simulated Annealing to improve the feature subset further:  ○Set the initial temperature T_0_ and cooling rate α.  ○At each iteration of Simulated Annealing, perform the following:
○Select a neighboring feature subset by flipping a random feature in *X*.
○Evaluate the fitness of the new subset.
○If the new subset has better fitness, accept it.
○If the new subset has worse fitness, accept it with a probability P = $e^{\frac{-∆}{T}}$, where Δf is the fitness difference and T is the current temperature.
○Decrease the temperature T = T·α.  ○After applying the local search, update the best feature subset found so far.**Step 4: Termination**Stop the algorithm when the maximum number of iterations T_max_ is reached or the solution converges (i.e., no significant improvement in fitness).The final output is the feature subset $X^{*}$(*t*) corresponding to the best fitness.**Step 5: Output**
Return the optimized feature subset $X^{*}$(*t*), representing the most significant features for classification.


### 2.4. Classification

The combination of forecasts from multiple fundamental models in stacking, also known as stacked generalization, is a powerful ensemble technique that enhances overall effectiveness. The classification of vitamin D levels into four classes is achieved by stacking in this study. Predictions from base classifiers are integrated by utilizing a Logistic Regression (LR) meta-classifier. To enhance a person’s health, it is essential to accurately identify these groups because of the diverse vitamin D classifications that necessitate specific care. Despite the use of bootstrap sampling and cross-validation for evaluating the performance of base learners, the combiner model’s exhibits are usually evaluated with a single test dataset. This approach could result in overly positive assessments due to the optimization of information.

Each base classifier is a diverse ML model that contributes unique insights to the stacking ensemble. Predictions were made using ML classifiers like Random Forest (RF), SVM, Naive Bayes (NB), K-Nearest Neighbor (KNN), extra Gradient Boosting (XGB), Decision Tree, and Gradient Boosting Machine (GBM), which also act as input features for the meta-classifier. The simplicity, interpretability, and effectiveness of LR as a meta-classifier make it a worthwhile choice for binary or multiclass classification tasks. LR provides a well-calibrated and computationally efficient approach because it is crucial to accurately differentiate between four levels of vitamin D in clinical decision-making. Moreover, this classifier is easy to understand, can handle probabilistic outputs efficiently, and has a lower risk of overfitting than neural networks and GB. Furthermore, it is easily adaptable to multiclass classification, which makes it suitable for real-time applications and medical contexts. The model’s performance, interpretability, and efficiency are balanced to maintain practical applicability in healthcare settings, ensuring a robust classification of vitamin D levels. Mathematically, the LR model is defined as

$$P(y=k|x)=\frac{exp(\beta_{k}^{T}x)}{\sum_{j=1}^{K}exp(\beta_{k}^{T}x)}$$

where the variables are as follows:

- P (y = *k*∣*x*) is the probability of class *k* given the feature vector *x*.
- $\beta_{k}\;$ represents the coefficients for class k.
- *K* is the total number of classes (in this case, four levels of vitamin D severity).

The collected VDD severity datasets underwent random partitioning into training (80%) and testing (20%) datasets. Various ML algorithms were employed on the training dataset. Following the training phase, where models were trained using the training dataset, the resulting models were utilized to predict deficiency severity on the testing dataset. To mitigate bias associated with the random dataset split, we conducted 10-fold cross-validation (CV) experiments, ensuring diverse samples in both training and testing datasets. The focal objective was to forecast the performance of a pivotal indicator within severity levels (1–4).

The confusion matrix of the ML classifiers that were used to analyze and compare individual models is displayed to evaluate model performance. The confusion matrix for different classification models gives valuable insight into their performance, particularly in terms of sample classification that is correct and incorrect. Various factors can lead to misclassification, such as overlapping feature distributions, model bias, insufficient training data, or inherent complexity in distinguishing certain categories. The step-by-step approach of LR stacking is defined in Algorithm 2.
**Algorithm 2:** LR stacking**Input:** Preprocessed dataset (X, y) containing 12 selected features and 4 VDD severity predicted levels
**Output:** Final predictions for the severity levels based on LRModel initiation (Base: RF, SVM, GB and Meta: LR)Data split with k-fold CVBase model training:
For each fold, split the data into training (X_train_, y_train_) and testing (X_test_, y_test_) sets and train each base model on X_train_, y_train_. Obtain predictions from each base model for X_test_ and store these predictions as meta-features for both X_train_ and X_test_.Construct Meta-Feature Matrix:
Combine the predictions of the base models into a new meta-feature matrix: X_meta_ = [RFC_pred_,SVM_pred_,GBC_pred_].Train LR on X_meta_ using the training fold.For the test set, pass the predictions from the base models through the trained LR to generate the final predictions.Model evaluation using performance metrics such as accuracy, precision, recall, F1-score, and confusion matrix.Perform steps 3–7 for all 10 folds and aggregate results to validate the model’s performance.Analysis of output predictions

### 2.5. Software and Hardware Requirements

Model training and evaluation were carried out using Python v3.12, and libraries like Scikit-learn, TensorFlow, and XGBoost were used. An Intel Core i7 processor and 16 GB of RAM (intel, Chandler, AZ, USA) were used to conduct experiments on a system that was running on Windows 11.

## 3. Results

### 3.1. Feature Correlation

**Figure 1:**
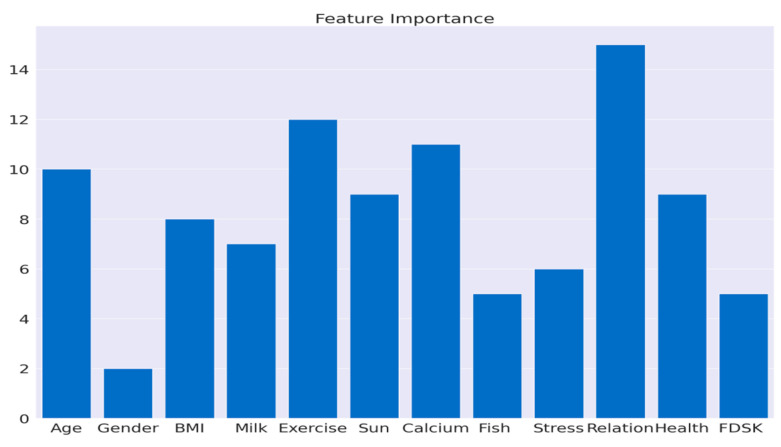
Score-based feature importance.

**Figure 2:**
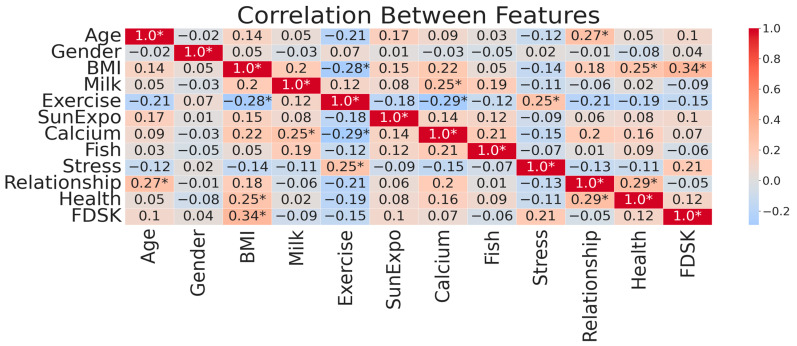
Feature correlation matrix (* |corr| > 0.25).

By analyzing feature importance, VDD can be predicted with valuable insights into the factors that influence the model’s outcomes. The importance of each feature is displayed in Figure 1 according to the scores. The correlation matrix in Figure 2 revealed the relationship between various features in the dataset. The correlation between age and BMI, sun exposure, and family relationships is weak to moderate, but there is a negative correlation with exercise habits. The correlation between gender and other features is minimal. The relationship between BMI and milk consumption, calcium intake, and family relationships is positive, while exercise habits and financial stress are negative. These values help us understand the interdependence of features through linear associations.

To make it easier to read, we emphasized strong correlations with an absolute value of the correlation coefficient (|corr|) exceeding 0.25 indicated with an asterisk (*) to these values. This guarantees that the key relationships between features are visible. In addition, we used an upper triangular mask to eliminate redundant values because correlation matrices have symmetry. A cleaner and more interpretable visualization is achieved by not displaying duplicate information. We effectively distinguished positive and negative correlations, and annotations are adjusted to improve clarity. Identifying significant correlations is made easier with the combination of these enhancements, while maintaining a structured and visually appealing presentation.

### 3.2. ML Model Performance

**Figure 3:**
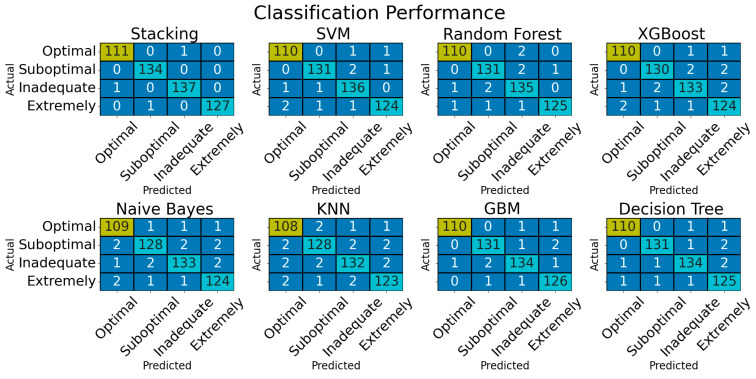
Confusion matrix outcomes of adopted models.

The confusion matrix of the ML classifiers used is depicted in Figure 3. The stacking model can perform well without any misclassifications, but it can occasionally misclassify samples between the ‘Optimal’, ‘Inadequate’, and ‘Extremely Inadequate’ categories. The base classifiers were struggling to capture subtle differences in feature values which could be the reason for this. Misclassification patterns in VDD detection are examined by various ML models including SVM [28], RF [29], XG Boost [30], NB [31], KNN [32], GBM [33], and DT [34] using different methodologies and assumptions. For instance, SVM struggles with overlapping feature distributions, while NB assumes feature independence, which leads to errors across all categories. By combing the strengths of multiple classifiers, the stacking model can prevent some of these errors.

Table 3 exhibits the overall performance of the proposed model. The model suggested exhibits impressive results at all levels of severity, attaining an accuracy of 1.00% in the ‘Suboptimal’ category, with precision, recall, and F1-score all being 99.26%, 1.00%, and 99.63%. With an accuracy of 99.10% and a precision, recall, and F1-score of 99.11, the Optimal category is considered subpar. The accuracy rates for the ’Inadequate’ and ’Extremely Inadequate’ categories are 99.27% and 99.21%, with high precision, recall, and F1-scores achieved. The SVM classifier performs well, with accuracy rates around 98% in different categories. The Decision Tree Classifier exhibits robust performance, with accuracy rates around 98% in various categories. The ‘Optimal’ class saw the Naive Bayes Classifier perform exceptionally well, with an accuracy of 97.32%. With a 98.21% accuracy, the RF Classifier excels in the ’Optimal’ category. The KNN Classifier and XG Boost Classifier maintained a high level of accuracy for all categories. The stacking model proposed is the best performer, with a 99.41% accuracy and remarkable predictive capabilities.

### 3.3. VDD Level’s Seasonal Variation

**Figure 4:**
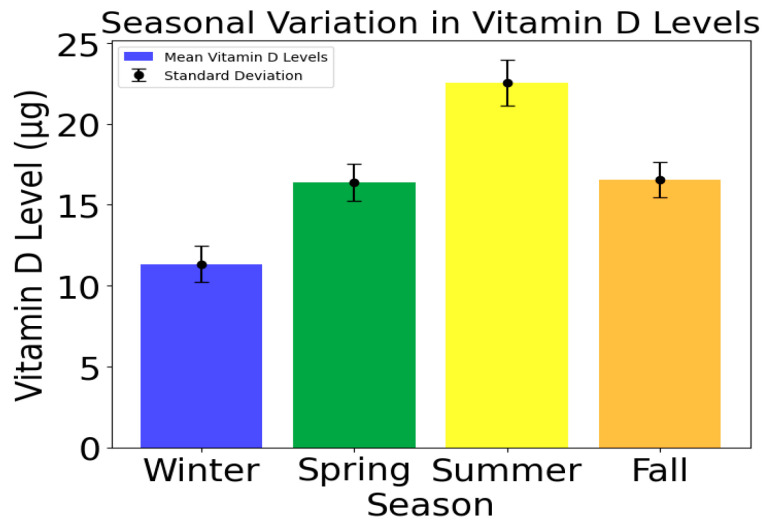
Seasonal variation.

VDD’s seasonal variation is noticeable, and its mean values peak at 22.55 during the summer, followed by a decline in the fall (16.56) and spring (16.38), as shown in Figure 4. The winter season has a mean vitamin D level of 11.33 which is the lowest. This seasonal fluctuation’s degree of variability is reflected in the standard deviations, which vary between 1.07 in the fall and 1.43 in the summer, suggesting that levels vary more during the warmer months.

**Figure 5:**
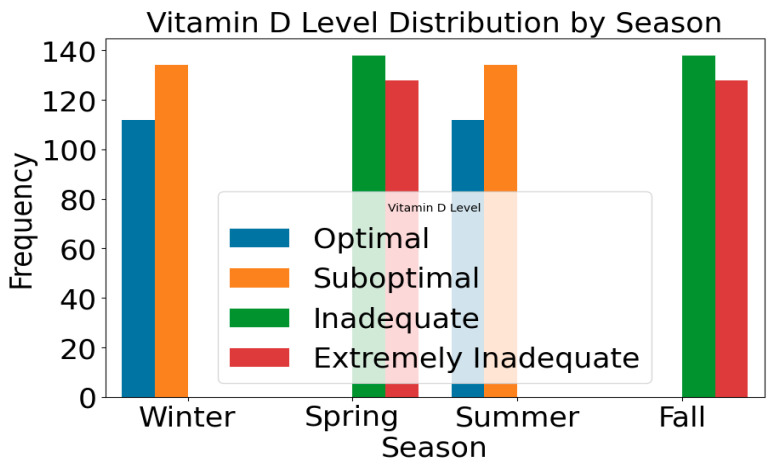
Frequency of participants within each level of severity.

The distribution of VDD levels during each season is shown in Figure 5. The distribution presents a valuable insight into the influence of seasonal variations on vitamin D levels among participants. Due to reduced sun exposure, certain seasons, such as winter, may show higher levels of Suboptimal and Inadequate vitamin D, while other seasons, such as summer, may show more Optimal levels. Vitamin D levels vary throughout the year due to factors such as sun exposure, dietary habits, and lifestyle choices, influencing overall vitamin D levels. The accuracy of health recommendations and interventions can be enhanced by tailoring predictive models to account for seasonal fluctuations after analyzing these seasonal patterns.

Normalization is a critical preprocessing step that adjusts data to a standard scale, ensuring that all features contribute equally to the analysis or model training. In this study, the 25-OH-D levels were normalized for each respective season. This transformation results in values that indicate the deviation of individual 25-OH-D levels from the seasonal mean and standard deviation (SD). This normalization process facilitates a clearer comparison of vitamin D levels across different seasons, removing seasonal bias and ensuring that the data are on a comparable scale, which is essential for effective analysis and model training. Table 4 tabulates the normalized values.

## 4. Discussion

In this paper, we propose a stacking classifier with an improved whale optimization for VDD prediction. The amount of ultraviolet B radiation that is available for vitamin D synthesis is influenced by variables such as the time of day, season, geographical location, and the use of sunscreen. Regular monitoring of vitamin D levels and appropriate supplementation when necessary are necessary due to factors such as age, skin pigmentation, and certain medications that can impact the body’s ability to produce vitamin D. Even though supplements are convenient, they cannot duplicate the holistic benefits that come from being exposed to natural sunlight and eating a well-rounded diet, and supplements should not be used as a substitute for these primary sources.

Individuals within each VDD level must ensure they attain the recommended vitamin D levels through a blend of exposure to natural sunlight, incorporating vitamin-rich foods into their diets, and possibly including supplements if necessary [35]. It should be underscored that an excessive intake of vitamin D can result in undesirable effects, including nausea, diminished appetite, vomiting, constipation, and weight loss [36]. Individuals with specific medical conditions, such as sarcoidosis, tuberculosis, lymphoma, and hyperparathyroidism, are strongly encouraged to seek guidance from a healthcare professional before considering vitamin D supplementation.

The model’s predictive power is increased by 15% due to familial relationships, which are the most influential factors in VDD, demonstrating the impact of family dynamics on dietary, outdoor, and health habits. Lifestyle, mental health, and social engagement can all play a role in influencing vitamin D levels through family relationships and overall happiness. Having strong family bonds and a happy environment can lead to better eating habits, regular outdoor activities, and lower stress levels, which are all important factors when it comes to adequate vitamin D levels. In contrast, those experiencing family conflicts or unhappiness may have more stress, depression, and social isolation, which can result in poor diet choices, decreased sun exposure, and an increased risk of VDD. According to studies [37,38], chronic stress and depression can cause disruptions in vitamin D metabolism, which can further worsen deficiency. The importance of maintaining optimal vitamin D levels lies in a supportive family environment and emotional well-being.

It is possible that the 12% importance given to exercise habits is due to increased exposure to the sun and its overall health benefits. Calcium intake and sun exposure played a role in vitamin D metabolism, with 11% and 9%, respectively. The age factor was 10%, which reflects age-related changes in synthesis. Moderate contributors were BMI (8%) and milk consumption (9%), while health issues (9%) and fish intake (5%) provided additional insights. The financial stress and FDSK scores (6% and 5%) indicated that dietary diversity was limited due to financial constraints, with gender having a minimal impact at 2%.

The correlations show that exercise habits have a negative correlation with BMI and health issues, while milk consumption has a positive association with calcium intake, strengthening the key interdependencies in predicting VDD. Besides, results demonstrated that sun exposure demonstrated a strong positive correlation with vitamin D levels, reinforcing the critical role of sunlight in vitamin D synthesis. Financial stress negatively correlated with milk consumption, fish intake, and FDSK Score, indicating that individuals under financial strain might compromise their dietary diversity and nutrient intake. Other features, such as age, gender, lifestyle, dietary habits, and health-related variables, exhibited nuanced relationships, providing valuable insights into the multifaceted contributors to VDD. This comprehensive visualization highlights the intricate interplay of factors influencing vitamin D levels and underscores the need for a holistic approach in addressing deficiency risks.

### 4.1. State-of-Art Literature

The performance of the proposed stacking classifier can be compared with the ensemble techniques used in state-of-the-art approaches, particularly in terms of accuracy, computational efficiency, and overall effectiveness. For instance, in [39], the Gradient Boosting method outperforms other algorithms in predicting heart disease, demonstrating high accuracy, recall, and F1-score. While Gradient Boosting is effective, it can be computationally intensive due to the iterative nature of boosting algorithms. Similarly, the study in [40] uses a stacking model with LogitBoost as the meta-classifier, where the final prediction is made after integrating four base classifiers. This approach also emphasizes accuracy but requires significant computational resources for training and evaluation.

The proposed stacking model in this research addresses both accuracy and computational efficiency by combining multiple base classifiers, such as Random Forest, Support Vector Machine, and Gradient Boosting, through a Logistic Regression meta-classifier. Logistic Regression is chosen specifically for its simplicity, interpretability, and computational efficiency compared to more complex models like neural networks or deep learning approaches, as seen in the study [41], where convolutional and deep neural networks are employed to extract features. These deep models, while accurate, are often more computationally expensive, requiring significant resources for both training and inference. In contrast, Logistic Regression, despite its simplicity, effectively combines the predictions from base classifiers, ensuring that the model remains lightweight and scalable without sacrificing too much accuracy.

In [42], a two-stage stacked method with models like Random Forests and extreme Gradient Boosting achieves a high accuracy (96%) and recall (98%). While the performance is strong, the model’s computational demands are likely to be higher due to the integration of multiple robust learners. The proposed stacking model, by contrast, offers a balance, ensuring a high performance while mitigating the risk of overfitting and reducing the computational burden associated with more complex models. Finally, the study in [43] combines SVM with XGBoost and utilizes feature selection techniques to enhance model performance. Although this results in improved accuracy, the combination of models and feature engineering increases the complexity of the model, which could lead to longer training times and higher resource consumption. The proposed stacking method, through its use of simpler classifiers (Random Forest, SVM, and Gradient Boosting) and a computationally efficient meta-classifier (Logistic Regression), ensures that accuracy is maintained without the significant computational cost seen in models relying on hyperparameter tuning or deep learning. The proposed stacking classifier achieves competitive accuracy while ensuring computational efficiency by leveraging the strengths of simpler base models and a Logistic Regression meta-classifier, making it a viable option in scenarios where real-time predictions and resource constraints are important.

### 4.2. Advantages of Stacking Classifier

The use of ML techniques to predict VDD severity has been explored by several studies, but this research stands out by using an IWOA method for feature selection and an improved stacking classifier for prediction. Traditional classification models, including Logistic Regression [44], Random Forest [45], and support vector regression [46], have been the focus of previous research, with no consideration for optimizing feature selection. While these studies showed a level of accuracy that was acceptable, they often failed to address issues with computational efficiency and overfitting. The proposed approach integrates feature selection and IWOA, which reduces feature redundancy and computational time while maintaining an accuracy rate of 99.40%. The methodology’s approach to VDD severity prediction is distinct due to the dual emphasis on precision and efficiency.

This study is different from previous studies that used limited datasets and fewer predictors and uses a comprehensive dataset of 512 participants and 12 diverse features, including lifestyle habits, dietary intake, and psychosocial factors. The features were meticulously curated and validated to provide a holistic perspective on the severity of VDD. Compared to studies that focused solely on clinical parameters like serum vitamin D levels, this work adopts a multifactorial approach that enriches the prediction process by including parameters such as sun exposure, family relationships, and financial stress. Conventional models are differentiated from this study by its broad-spectrum analysis, which provides a more detailed understanding of VDD severity.

This research is further distinguished using a stacking classifier. Despite the use of single-model approaches in previous studies [47], the ensemble technique [48] utilizes the collective strength of multiple classifiers to create a more robust and generalized prediction system. By using probabilistic sampling strategies and strong samples in building stacking classifiers, model stability and generalizability are enhanced. With this comprehensive framework, healthcare professionals can identify and manage VDD with greater precision by accurately predicting four nuanced severity levels, which is a practical solution.

### 4.3. Limitations

Despite the improvements made in this study, there are still some limitations that need to be addressed. By gathering larger, more varied datasets and incorporating additional predictive features, future work could overcome these limitations. Integrating the model into clinical workflows and validating its performance in real-world medical settings would be an essential following step. To improve the system’s predictive capabilities, research could explore hybrid optimization techniques and adaptive models, leading to its use in more healthcare applications.

## 5. Conclusions

Our work focused on proposing the most efficient ML model for predicting the severity of VDD. The accuracy of the prediction was assessed by thoroughly comparing training and testing sets. The study evaluated key performance metrics such as precision, recall, F1-score, and accuracy using six different machine learning models. An improved whale optimization technique was employed to select 12 features for the severity prediction. ML methodologies have been demonstrated to be viable alternatives for accurately predicting VDD severity. In accordance with the research findings, machine learning models, particularly stacking classifiers, display remarkable accuracy in predicting the severity of VDD. Furthermore, the proposed classification system attained the highest accuracy of 99.4%, which surpasses the performance of the other classification systems. The significance of this classifier in medical applications lies in its ability to assist experts in efficiently identifying the severity of VDD. This study offers a significant advantage by incorporating a novel approach to predicting VDD severity, along with an introduced classifier and comprehensive evaluation using diverse performance metrics. The study states that the proposed classifier is more effective in predicting the severity of vitamin D deficiency than other models.

## Figures and Tables

**Table 1:** Dataset features.

Feature	Values
Age	<40, 40–50, >=50
Gender	Male/Female
BMI	<25 kg/m^2^, >=25 kg/m^2^
Milk Consumption	Yes or no
Exercise Habit	Yes or no
Sun Exposure	Always, Often, Sometimes, Infrequently, Never
Calcium Intake Score	>16, 1–15, 0–7
Fish Intake	Yes/No
Financial Stress	Yes/No
Relationship with Family	Happy/Unhappy
Geographic Location	Urban, Rural
Air Quality Index	Good, Moderate, Poor, Very Poor
Health Issues	Yes/No
FDSK Score	Score value

**Table 2:** Severity-level distribution in the dataset.

Severity	Description	Frequency	Percentage (%)
Level 1	Optimal	112	21.88
Level 2	Suboptimal	134	26.17
Level 3	Inadequate	138	26.95
Level 4	Extremely Inadequate	128	25.00

**Table 3:** Classification performance.

Classifier	Accuracy	Precision	Recall	F1-Score	Class
Proposed	0.9910	0.9911	0.9911	0.9911	Optimal
	1.0	0.9926	1.0000	0.9963	Suboptimal
	0.9927	0.9928	0.9928	0.9928	Inadequate
	0.9921	1.0000	0.9922	0.9961	Extremely Inadequate
SVM [28]	0.9821	0.9735	0.9821	0.9778	Optimal
	0.9776	0.9850	0.9776	0.9813	Suboptimal
	0.9855	0.9714	0.9855	0.9784	Inadequate
	0.9687	0.9841	0.9688	0.9764	Extremely Inadequate
RF [29]	0.9821	0.9821	0.9821	0.9821	Optimal
	0.9776	0.9776	0.9776	0.9776	Suboptimal
	0.9782	0.9643	0.9783	0.9712	Inadequate
	0.9765	0.9921	0.9766	0.9843	Extremely Inadequate
XGBoost [30]	0.9821	0.9735	0.9821	0.9778	Optimal
	0.9701	0.9774	0.9701	0.9738	Suboptimal
	0.9637	0.9708	0.9638	0.9673	Inadequate
	0.9687	0.9612	0.9688	0.9650	Extremely Inadequate
NB [31]	0.9732	0.9561	0.9732	0.9646	Optimal
	0.9552	0.9697	0.9552	0.9624	Suboptimal
	0.9637	0.9708	0.9638	0.9673	Inadequate
	0.9687	0.9612	0.9688	0.9650	Extremely Inadequate
KNN [32]	0.9642	0.9474	0.9643	0.9558	Optimal
	0.9552	0.9624	0.9552	0.9588	Suboptimal
	0.9565	0.9635	0.9565	0.9600	Inadequate
	0.9609	0.9609	0.9609	0.9609	Extremely Inadequate
GBM [33]	0.9821	0.9910	0.9821	0.9865	Optimal
	0.9776	0.9776	0.9776	0.9776	Suboptimal
	0.9710	0.9781	0.9710	0.9745	Inadequate
	0.9843	0.9692	0.9844	0.9767	Extremely Inadequate
Decision Tree [34]	0.9821	0.9821	0.9821	0.9821	Optimal
	0.9776	0.9850	0.9776	0.9813	Suboptimal
	0.9710	0.9781	0.9710	0.9745	Inadequate

**Table 4:** Vitamin D levels.

Season	25-OH-D Level (Mean ± SD)	Normalized 25-OH-D Level (Mean ± SD)
Winter	12.5 ± 1.5	−0.75 ± 1.50
Spring	18.5 ± 1.5	−0.75 ± 1.50
Summer	22.0 ± 2.0	−1.00 ± 2.00
Fall	15.0 ± 1.5	0.75 ± 1.50

## Data Availability

Availability of data can be done based on personal requests.
